# Development of a Clinical and Genetic Prediction Model for Early Intestinal Resection in Patients with Crohn’s Disease: Results from the IMPACT Study

**DOI:** 10.3390/jcm10040633

**Published:** 2021-02-07

**Authors:** Eun Ae Kang, Jongha Jang, Chang Hwan Choi, Sang Bum Kang, Ki Bae Bang, Tae Oh Kim, Geom Seog Seo, Jae Myung Cha, Jaeyoung Chun, Yunho Jung, Hyun Gun Kim, Jong Pil Im, Sangsoo Kim, Kwang Sung Ahn, Chang Kyun Lee, Hyo Jong Kim, Min Suk Kim, Dong Il Park

**Affiliations:** 1 Department of Internal Medicine, Institute of Gastroenterology, Yonsei University College of Medicine, Seoul 03722, Korea; EAKang@yuhs.ac; 2 Department of Bioinformatics, Soongsil University, Seoul 06978, Korea; whdgkwkd@gmail.com (J.J.); sskimb@gmail.com (S.K.); 3 Department of Internal Medicine, College of Medicine, Chung-Ang University, Seoul 06978, Korea; gicch@cau.ac.kr; 4 Department of Internal Medicine, College of Medicine, Daejeon St. Mary’s Hospital, The Catholic University of Korea, Daejeon 34943, Korea; sangucsd@gmail.com; 5 Department of Internal Medicine, Dankook University College of Medicine, Cheonan 31116, Korea; kibaebang@gmail.com; 6 Department of Internal Medicine, Haeundae Paik Hospital, Inje University College of Medicine, Busan 48108, Korea; kto0440@paik.ac.kr; 7 Department of Internal Medicine, Digestive Disease Research Institute, Wonkwang University College of Medicine, Iksan 54538, Korea; medsgs@wku.ac.kr; 8 Department of Internal Medicine, Kyung Hee University Hospital at Gang Dong, Kyung Hee University College of Medicine, Seoul 05278, Korea; clicknox@hanmail.net; 9 Department of Internal Medicine, Gangnam Severance Hospital, Yonsei University College of Medicine, Seoul 06273, Korea; j40479@gmail.com; 10 Department of Internal Medicine, Soonchunhyang University College of Medicine, Chungnam 31151, Korea; yoonho7575@naver.com; 11 Department of Internal Medicine, Soonchunhyang University College of Medicine, Seoul 04401, Korea; medgun@schmc.ac.kr; 12 Department of Internal Medicine and Liver Research Institute, College of Medicine, Seoul National University, Seoul 03080, Korea; jpim0911@snu.ac.kr; 13 Functional Genome Institute, PDXen Biosystems Inc., Seoul 34129, Korea; kwangsung.ahn@gmail.com; 14 Department of Internal Medicine, Kyunghee University School of Medicine, Seoul 02454, Korea; gidrlee@gmail.com (C.K.L.); hjkim@khmc.or.kr (H.J.K.); 15 Department of Human Intelligence and Robot Engineering, Sangmyung University, Chungcheongnam-do 31066, Korea; minsuk.kim@smu.ac.kr; 16 Division of Gastroenterology, Department of Internal Medicine and Gastrointestinal Cancer Center, Kangbuk Samsung Hospital, Sungkyunkwan University School of Medicine, Seoul 03181, Korea

**Keywords:** Crohn’s disease, machine learning, genetic variation, prognosis, surgery

## Abstract

Early intestinal resection in patients with Crohn’s disease (CD) is necessary due to a severe and complicating disease course. Herein, we aim to predict which patients with CD need early intestinal resection within 3 years of diagnosis, according to a tree-based machine learning technique. The single-nucleotide polymorphism (SNP) genotype data for 337 CD patients recruited from 15 hospitals were typed using the Korea Biobank Array. For external validation, an additional 126 CD patients were genotyped. The predictive model was trained using the 102 candidate SNPs and seven sets of clinical information (age, sex, cigarette smoking, disease location, disease behavior, upper gastrointestinal involvement, and perianal disease) by employing a tree-based machine learning method (CatBoost). The importance of each feature was measured using the Shapley Additive Explanations (SHAP) model. The final model comprised two clinical parameters (age and disease behavior) and four SNPs (rs28785174, rs60532570, rs13056955, and rs7660164). The combined clinical–genetic model predicted early surgery more accurately than a clinical-only model in both internal (area under the receiver operating characteristic (AUROC), 0.878 vs. 0.782; *n* = 51; *p* < 0.001) and external validation (AUROC, 0.836 vs. 0.805; *n* = 126; *p* < 0.001). Identification of genetic polymorphisms and clinical features enhanced the prediction of early intestinal resection in patients with CD.

## 1. Introduction

Crohn’s disease (CD) is one of the main forms of inflammatory bowel disease (IBD), and it is characterized by chronic, relapsing inflammation of the entire gastrointestinal tract. CD can occur as a result of interactions among multiple factors, including genetic predisposition, immunologic dysregulation, alterations in gut microbiome, and environmental factors [1]. Previous studies discovered over 140 genetic mutations related to CD including single-nucleotide polymorphisms (SNPs) within Nucleotide-binding oligomerization domain-containing protein 2 (NOD2), Interleukin-23 receptor (IL23R), Janus kinase 2 (JAK2), Autophagy related 16 like 1 (ATG16L1), Immunity-related GTPase family M (IRGM), and Signal transducer and activator of transcription 3 (STAT3) [2,3,4,5].

CD occurs mainly at a young age, causing various complications ultimately reducing the patient’s quality of life. Many complications arising from the disease courses of CD may require intestinal resection. Early intestinal resection is required in complicated CD, which does not improve with medical treatment. In a previous study, early surgery in CD was defined as major surgery within 3 years of diagnosis [6]. The need for early intestinal resection after diagnosis of CD results in a poor prognosis [7,8]. Due to the complexity of the disease process in each CD patient, personalized treatment is required. However, it is difficult to predict disease course and select patients who are at high risk of early intestinal resection. Therefore, it is important to predict and screen CD patients with a high risk of early intestinal resection, because disease exacerbation can be prevented by intensified therapy, such as an early combination therapy with biologics [9,10].

Previous studies reported several clinical and genetic factors related to the risk of early intestinal resection in patients with CD. Response to biologics and a complicated disease course are risk factors for early intestinal resection in CD [11]. Age at diagnosis, disease behavior, jejunum involvement, and perianal disease are associated with a poor prognosis of CD [7]. Previous studies have suggested that bowel wall thickening, according to an abdominal ultrasound, is associated with a 1-year risk of surgery in patients with CD [12,13]. Clinical prediction models have been suggested for surgery and complications in CD using clinical trial data; however, their prediction power is low [14]. In order to minimize the diversity and judgment of the clinical characteristics of Crohn’s disease, which may vary from institution to institution, it is necessary to develop a predictive model with the addition of genetic data. To the best of our knowledge, a risk scoring system or predictive model for early surgery in CD using genetic factors has not been previously reported. Here, we suggest a prediction model for early intestinal resection within 3 years of diagnosis of CD, according to clinical and genetic data of patients with CD using machine learning. For machine learning, we employed CatBoost, an ensemble tree-based algorithm that can conveniently handle both categorical and numerical features [15,16].

## 2. Materials and Methods

### 2.1. Study Population

Patients with CD were included from 15 tertiary hospitals in South Korea that participated in the multicenter, retrospective case-control study (IMPACT study: identification of the mechanism of the occurrence and progression of Crohn’s disease through integrated analysis on both genetic and environmental factors). Diagnosis of CD was based on comprehensive analysis of the clinical manifestation, endoscopic findings, histopathological findings, radiologic images, and serologic markers. Clinical information, including age, sex, cigarette smoking, family history of IBD, disease location, and behavior at diagnosis, categorized according to the Montreal classification, was analyzed [17]. Blood samples for genetic analysis were obtained from all patients enrolled in this study. We initially enrolled patients with CD with both clinical and genotype data from May 2017 to May 2020. Furthermore, we excluded patients who were diagnosed within the last 3 years.

### 2.2. Genotyping

CD patients enrolled in this study were genotyped using the Korea Biobank Array [18], which is available for genetic studies in the Korean population and contains 833,000 SNPs. For the genome-wide association study (GWAS), we conducted sample quality control (QC) and data imputation to increase the statistical significance in geno/phenotype and case/control analysis [19,20,21]. Sample QC filtered out abnormal samples through call rate, heterozygote, and relative relationship tests provided by PLINK, which is a tool set for whole-genome data analysis [22]. An additional principal component analysis was conducted, and outliers were filtered. Markers with minor allele frequency less than 1%, Hardy Weinberg equilibrium *p*-value <1 × 10^−5^, and 0.1% missing SNPs were removed. Following this process, 544 samples and 625,238 SNPs remained. Additionally, we conducted an imputation process to complete the information regarding SNPs. To increase the accuracy and stability of imputation, the Korea Biobank Array genotype data of 27,545 normal individuals generated by the Korea National Institute of Health were added. Using a Michigan imputation server and corresponding parameters, we yielded a genotype set of 6,228,601 SNPs.

We aimed to identify candidate SNPs using the GWAS as a prescreening method. For the case/control GWAS, the commonly used single-scan method was employed. Each SNP was scanned sequentially, using the null hypothesis of no association. PLINK supports five different genetic models (allelic, dominant, recessive, genotypic, and trend) to test the SNPs associated with the phenotype. Candidate SNPs were selected via five independent tests with a threshold *p*-value <2 × 10^−6^. The threshold *p*-value was set higher than the genome-wide significance (5 × 10^−8^) to see a combination of SNPs by securing the genotype diversity. The SNP genotype was encoded as a categorical variable regardless of its genetic origin.

### 2.3. Machine Learning for a Prediction Model

We combined the genotype and clinical information of candidate SNPs, obtained from GWAS, for machine learning analysis using the CatBoost algorithm [23]. Since it is a tree-based model, data normalization is not necessary and categorical variables do not need to be preprocessed [15]. CatBoost is a model that considers the correlation of features and performs well with data containing categorical variables. CatBoost was used via the Python package.

The contribution of each feature to the model prediction was assessed using the Shapley Additive Explanations (SHAP) approach, which ensures high local accuracy, stability against missing data, and consistency in feature impact [24]. The SHAP values were calculated using TreeSHAP [25]. To avoid overfitting issues, the features with the lowest SHAP values were successively eliminated, while the area under the receiver operating characteristic (AUROC) at each step was not lower by 0.05 than the fivefold cross-validation (CV) AUROC of the initial model.

### 2.4. Performance Evaluation

Combining clinical information and genotype data, after excluding patients who were diagnosed with CD less than 3 years prior, 337 patients remained and were used as the discovery cohort for machine learning. Of these, 46 patients (14%) had intestinal resection within 3 years of CD diagnosis (cases), and the remaining 291 patients had no intestinal resection during that period (controls). The discovery set (*n* = 337) was further divided into the training (*n* = 286) and internal validation sets (*n* = 51), with random allocation (85:15). For external validation, an additional 126 patients with CD were recruited from an independent hospital and genotyped using the same platform; 19 (15.0%) of the 126 patients underwent intestinal resection within 3 years of diagnosis with CD (Table S1, Supplementary Materials).

The performance of a prediction model was measured using AUROC [26]. To maximize the amount of data, we performed fivefold CV in the training set to identify the optimal hyperparameters through Bayesian optimization and compared the training models through fivefold CV AUROC. XGBoost [27], Gaussian naïve Bayes, random forest [28], and logistic regression were used for further model evaluation and comparison. We used the Python package to run the model.

## 3. Results

### 3.1. Baseline Characteristics of Study Population

We enrolled 439 patients with both clinical and genotype data, and then excluded patients who were diagnosed less than 3 years ago. Finally, clinical and genetic data of 337 patients were obtained. As mentioned above, 46 patients (13.6%) experienced intestinal resection within 3 years of CD diagnosis (cases). The mean ages of early surgery group and controls were 42.7 and 36.4 years, respectively, and the mean follow-up duration was 6.43 and 9.09 years, respectively. The early surgical group was older and had relatively short follow-up durations; the rate of stricturing disease at diagnosis was also higher than that of the control group. Details of baseline characteristics are shown in Table 1. Baseline clinical characteristics for each training set and internal validation set are shown in Table S2 (Supplementary Materials).

### 3.2. Candidate SNPs

The 6,228,601 imputed SNPs were prescreened using GWAS against the training set (37 cases and 249 controls) for inclusion in the prediction model. A total of 102 SNPs passed the threshold of *p*-value of 2 × 10^−6^; the recessive, trend, and allelic tests gave rise to 14, 76, and 12 nonredundant SNPs, respectively. The genotypic and dominant tests did not produce either nonredundant or significant results. As dependencies between features are less of a concern in CatBoost, linkage disequilibrium (LD) clumping was not attempted, unless the LD exceeded 0.85 (*r^2^*).

### 3.3. Selection of Clinical and SNP Features Using CatBoost and SHAP Values

**Figure 1:**
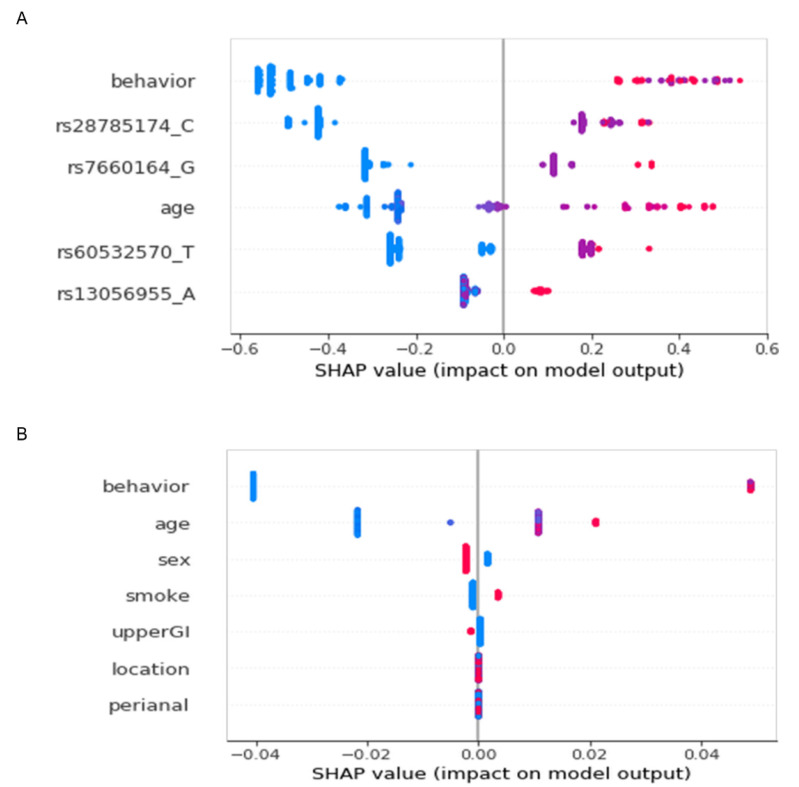
Shapley Additive Explanations (SHAP) summary plot by TreeSHAP. The features are shown in descending order of the mean absolute SHAP value on the *Y*-axis. Each dot represents a feature SHAP value of a patient. Categorical feature values are distinguished by color. Genotype and disease behavior are divided into three categorical features and represented by blue (major homozygote or noncomplicated), purple (heterozygote or stricturing), and red (minor homozygote or penetrating) colors. Age, a continuous variable, is represented by a color gradient from blue (young) to red (old). Features which are divided into two categories are represented by blue and red colors. (**A**) The final model comprising six clinical and genetic features. (**B**) The model based on seven clinical features only.

The initial model was trained using CatBoost with 102 SNP genotypes as features against the training set and the following seven clinical parameters: age, sex, cigarette smoking, disease location, disease behavior at diagnosis, upper gastrointestinal involvement, and perianal disease. The hyperparameters were tuned via Bayesian optimization through fivefold CV of the training set, and the average fivefold CV AUROC was 0.976. The internal validation AUROC was 0.552; the large discrepancy implied overfitting of this model. The importance of the features was evaluated by SHAP values obtained from TreeSHAP. A total of 20 rounds of feature selection yielded six features (Table S3, Supplementary Materials). Among the seven clinical parameters, age and disease behavior were selected, and four out of 102 SNPs remained. The impact of each variable on the model output was evaluated by the SHAP summary plot, which showed that behavior had the largest contribution to the prediction model, followed by age, and the four SNPs (Figure 1). The fivefold CV AUROC of the model with these six features was 0.929 ± 0.056, while the internal and external validation AUROCs were 0.878 and 0.835, respectively. With the internal set prediction probability cutoff of 0.5, the sensitivity and specificity were 0.62 and 0.95, respectively. the external set prediction probability cutoff of 0.5 showed that sensitivity and specificity were 0.84 and 0.72, respectively. In addition, the four SNPs were independent of behavior, the most important clinical feature in the model (nonlinear correlation with *p* < 0.01).

### 3.4. Contribution of Genotypes in Model Performance Compared to Clinical-Only Model

**Figure 2:**
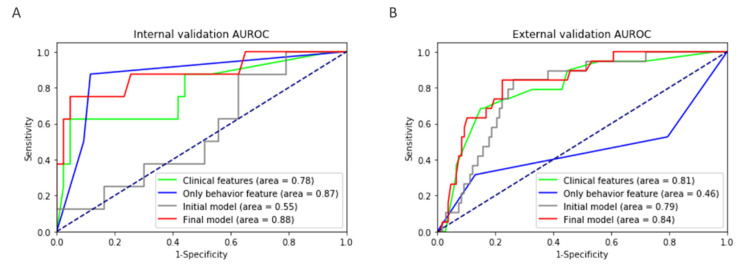
Comparison of four different feature combinations. The (**A**) internal and (**B**) external validation area under the receiver operating characteristic (AUROC) plots.

To evaluate whether the addition of genotypes improved the prediction performance over clinical information alone, a new CatBoost model using only clinical information was developed using the initial seven clinical features through a similar process, but without feature elimination. The resulting internal and external validation AUROCs were 0.782 and 0.805, respectively, which were less than those of the combined clinical and genetic model (Table 2, Figure 2). This clearly indicated that the addition of genotypes into the model improved its performance. The SHAP value summary plots (Figure 1) showed that the importance of behavior remained the highest, regardless of the addition of genotypes. The contribution of the other five clinical features, sex, smoking, location, upper gastrointestinal tract involvement, and perianal disease, diminished substantially upon the addition of genotypes.

### 3.5. Comparison between Our Prediction Model and Well-Known Machine Learning Models

**Figure 3:**
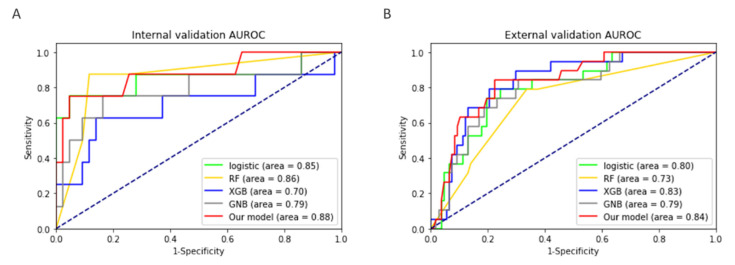
Comparison of performance of well-known machine learning models. The (**A**) internal and (**B**) external validation areas under the receiver operating characteristic (AUROC) plots. GNB, Gaussian naïve Bayes; RF, random forest; XGB, XGBoost.

We also measured performance using other well-known machine learning methods to compare the performance of the present model. For evaluation, one-hot encoding of the categorical variable behavior was processed. The performance of our model was the highest in both validation tests (Figure 3). In most models, if one validation set was high, the other was low; however, our model showed balanced results in both validation sets. All the models, except logistic regression, were tuned to hyperparameters via Bayesian optimization.

### 3.6. Risk Prediction of Intestinal Resection According to the Timing of Surgery

We estimated the sensitivity of the model according to the timing of surgery. In addition to the early intestinal resection within 3 years of diagnosis, the predictive performance of the model was further analyzed by dividing the timing of surgery into within 1 year, within 5 years, and any period after diagnosis. The probability of intestinal resection was measured using the final model and clinical-only model. AUROC values of internal and external validation according to the timing of surgery are shown in Table 3. The model’s predictive performance substantially improved when evaluating the risk of undergoing intestinal resection earlier after diagnosis, especially within 1 year. The predictive performance decreased as the period from diagnosis to surgery increased. The performance of the final model was better than the clinical-only model, but the superiority of the final model over the clinical-only model decreased as the criteria for the timing of surgery increased. Therefore, our model showed great predictive performance of intestinal resection within 3 years, especially within 1 year of CD diagnosis.

**Figure 4:**
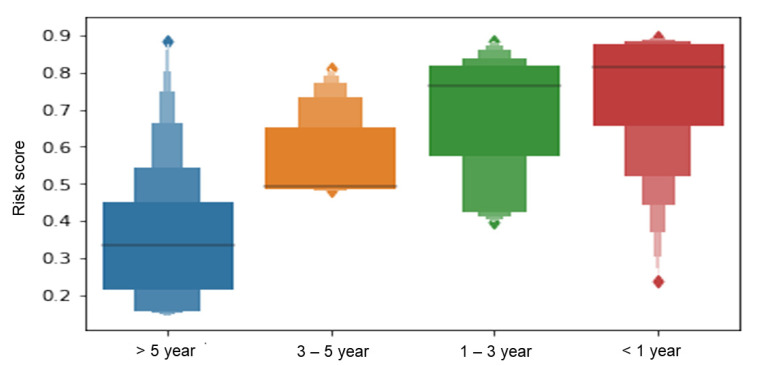
The Boxen plot of case probability by criteria of early intestinal resection using a training set of the final model. The case probability is expressed in the form of a Boxen plot.

We also checked the case probability of training set according to the criteria of each period to explain how the final model learned (Figure 4). The case probabilities were results of machine learning between 0 and 1. A higher score of the case probability denotes a higher risk of early intestinal resection. As a result, out final model showed that a longer duration to surgery led to a lower case probability.

### 3.7. Personalized Prediction of Intestinal Resection in Each Patient

**Figure 5:**
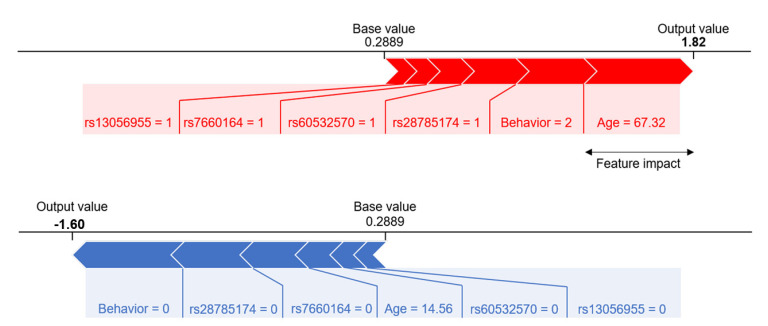
Representative SHAP force plots showing patient profiles of feature importance. Two extreme patients having the highest (upper panel) and lowest (lower panel) model probabilities were selected from the training set. The features are ordered by absolute SHAP value, which is represented by the arrow width. The corresponding feature profile is provided below the bar plot, where “output” and “base” values represent the patient SHAP values and the mean thereof, respectively.

For a particular prediction, the importance of each feature was assessed using the SHAP value framework. One of the benefits of evaluating feature importance on the basis of the SHAP value framework is its local interpretability. Two cases of patients with CD were examined to evaluate prediction probabilities. Firstly, for a 67-year old patient with penetrating CD, the incidence of intestinal resection within 3 years of diagnosis calculated by the model was approximately 86% (Figure 5). The model explained that all six features contributed toward the risk of intestine resection. Interestingly, in this case, age had a larger contribution than behavior, while behavior globally contributed more than age. Secondly, in a 14-year old CD patient without complications, the predictive probability of intestinal resection was 16.8%. All six variables were below the risk value of intestinal resection.

## 4. Discussion

The combination of CatBoost and SHAP models provided an accurate prediction of the risk of early intestinal resection in CD patients. A total of six features were used in the prediction model, including two clinical characteristics (behavior and age) and four SNPs. Adding genetic variation to the prediction model improved its predictive power compared with models that used only clinical features. Our final model showed higher predictive power both in internal and external validation sets than other models, such as logistic regression, random forest, XGBoost, and Gaussian naïve Bayes. Furthermore, the visualized SHAP plot showed the importance of each factor in CatBoost and provided individualized significance for each of the six characteristics per patient. In the future, this model may enable a personalized treatment approach for CD patients with a high risk of early intestinal resection and poor prognosis.

Due to the nature of CD, which chronically recurs and causes various complications in the abdominal cavity, intestinal resection is inevitable in most CD cases during the disease process. Pooled risk of surgery within 1, 5, and 10 years was reported as 14.3%, 27.7%, and 38.7% of patients diagnosed with CD since 1990, respectively. Even after the year 2000, the risk of surgery within 5 years was 24.2% [29]. Predicting the risk of early surgery allows optimal and patient-specific treatment. Several studies have suggested risk factors or prediction models for early surgery in CD. One study suggested a combined clinical-, endoscopic-, and sonographic-based risk matrix model for estimating the 1-year risk of surgery [30]. In the aforementioned study, disease behavior increased the risk of surgery independently (odds ratio (OR), 4.3; *p* < 0.001). Another study also investigated the risk for CD-related 1 year surgery. Disease behavior, smoking, body mass index, C-reactive protein (CRP), previous surgery, use of biologics, and enteral nutrition were associated with the risk of surgery [31]. On the basis of a retrospective cohort study, the Lémann index, an image-based measure for structural damage in CD, could help predict the risk of surgery within 1 year in CD [32]. In general, the behavior of CD at diagnosis has been identified as an independent risk factor for early surgery. Particularly, in ileal CD with stricture, prestenotic dilatation, CRP, combined penetrating behavior, exposure to biologics, and presence of NOD2 rs2066844 risk allele are all significantly associated with an increased risk of surgery [33]. However, no study has been performed using machine learning techniques to develop prediction models regarding surgery risk using combined clinical and genetic data. Our clinical-only model showed moderate AUROCs of 0.782 (internal validation) and 0.805 (external validation), similar to previous studies.

As mentioned above, the behavioral variable representing the state of the intestine had the greatest influence on the model. However, behavior alone exhibited highly biased performance in the external validation (AUROC_,_ 0.457). We assumed that the AUROC difference in the behavior-only model between the internal and external validation sets was due to the difference in disease duration (Table 1). This is because a longer disease period leads to a greater likelihood that the disease behavior will develop into a stricturing or penetrating disease. When other clinical variables were added, the bias was mitigated substantially (AUROC, 0.805). A CatBoost model with genotypes only and without any clinical parameters did not perform well. Albeit small by themselves, adding the genetic factors into the model had a profound effect in terms of performance. The final model had higher predictive power in external validation (AUROC, 0.835) than the model with only clinical characteristics. Taken together, the prediction model that incorporates genotype can improve the predictive power and minimize the use of clinical parameters that may vary among hospitals.

Among the four selected SNPs, the most important SNPs, rs28785174 (OR, 4.8; *p* = 1 × 10^−7^) and rs7660167 (OR, 4.7; *p* = 2 × 10^−7^), are located approximately 2 kb apart from each other on chromosome 4 and exhibit strong LD (*r^2^* = 0.83). They are located in the intronic region of follistatin-like 5 (FSTL5), a protein-coding gene that interacts with a metalloproteinase at the extracellular matrix level and is annotated with the calcium ion-binding gene ontology. FSTL5 is associated with diseases such as medulloblastoma, schizophrenia, hepatocellular carcinoma, and colorectal cancer [34,35,36,37,38]. In a multicenter study conducted in Spain, FSTL5 was associated with susceptibility to bone marrow suppression after thiopurine treatment in IBD patients [39]. Matrix metalloproteinase (MMP) levels are elevated in IBD [40], and increased levels of MMP are likely to contribute toward colonic tissue damage and recovery [41]. Intestinal fibrosis and stricture can be caused by an excessive deposition of extracellular matrix through the suppression of MMP [42]. According to the relationship between FSTL5 and MMP, we can assume that the new SNP associated with FSTL5 is related to the stricture behavior of CD.

The third important SNP, rs60532570 (OR, 4.4; *p* = 2 × 10^−6^), is located in the intronic region of Growth Factor Receptor Bound Protein 10-interacting GYF protein 2 (GIGYF2) gene on chromosome 2, and it is known to regulate the alternative splicing of GIGYF2 in fibroblast cell lines and thyroid. GIGYF2 is a protein-coding gene involved in the regulation of tyrosine kinase receptor signals. Diseases associated with this gene include Parkinson’s disease (PD) [43]. There are numerous studies indicating a link between PD and CD, which suggests that the GIGYF2 gene may be the link between the two diseases. The last SNP, rs13056955 (OR, 6.6; *p* = 3 × 10^−7^), is located more than 50 kb away from any known RefSeq gene on chromosome 22, and it is currently difficult to describe its biological significance. Nevertheless, mutations in the four SNPs in the prediction model are likely to increase the risk of early surgery within 3 years, due to a poor prognosis for CD.

CatBoost has high performance when there are categorical variables within the data, and it has the advantage of not requiring preprocessing. Moreover, well-known models, such as XGBoost, Gaussian naïve Bayes, random forest, and logistic regression, require preprocessing for categorical variables. Using CatBoost, we treated most of our clinical parameters and all unordered genotypes categorically. Although the SNPs originated from several GWAS runs of diverse genetic modes, they were incorporated into the model simultaneously, without preprocessing. Three of the four SNPs in our final model had trendy associations, while the other (rs13056955) had a recessive association [19]. For example, a patient with major homozygous or heterozygous rs13056955 showed SHAP values similar to each other but distinct from the minor homozygotes (Figure 1). This demonstrates that CatBoost is a powerful and flexible tool for combining clinical and genetic factors. However, the allele found in a previous study was not significant in our results, such as rs2066844 [33]. This study was conducted on Korean patients only and showed relevant SNPs specific to Korea. Further research is needed due to the heterogeneity of SNPs between ethnicities and races.

Our final model can predict the probability of early intestinal resection without a defined cutoff. Although we did not include a period from diagnosis to intestinal resection surgery in the learning process, the final model calculated the highest probability for patients who operated within 1 year and as the period increased, a probability decreased (Figure 4). This means that our final model is very specialized in predicting risk of early intestinal resection.

While machine learning algorithms are capable of solving complex problems, such as clinical data mining with high predictive power, it is difficult to explain how exactly the decision processes work. For example, tree-based models, such as CatBoost and random forest, typically generate dozens to hundreds of trees to solve the problem. However, it is almost impossible to manually comprehend how the features are used. In this case, the SHAP model is effective, as it summarizes the overall impact of the tree ensemble as the relative contribution of each feature to the classification of a specific sample.

Our study has a few limitations. First, although we conducted a multicenter study, there were shortcomings due to the small number of enrolled patients. The rate of intestinal resection within 3 years was lower (13%) than that reported by previous studies [29,30,31,44]. We performed fivefold CV to overcome the small sample size and possible data imbalance and used AUROC. We also tried to overcome it through external validation, which involved completely different hospital data that were not used for training separately from internal validation. Second, there may have been bias in chip array data. We used Korea Biobank Array genotype data for our GWAS; thus, we may have problems applying this method to other ethnicities. Accordingly, further studies using this prediction model with other population-specific genotyping arrays are warranted. We could not rule out the effects of early biologics or anti-Tumor necrosis factor (TNF) agents on clinical outcomes. Early use of anti-TNF and other biologics may have a protective effect for early intestinal resection. However, it is difficult to say that the use of anti-TNF agents affected the outcome because most anti-TNF treatments started before surgery, and the ratio of using anti-TNF agents also did not differ between the group that underwent intestinal resection (50.0%) and the group that did not (51.9%).

## 5. Conclusions

In conclusion, this new model, which includes clinical and genetic factors using the CatBoost machine learning technique and SHAP method, can predict the risk of early intestinal resection within 3 years of diagnosis with CD. The prediction requires only two clinical features (age, behavior) and four SNPs, and the importance of every feature can be individually estimated in each patient. Therefore, this model could allow the screening of CD patients at high risk for surgery and improve patient outcomes through proactive treatment and active surveillance.

## Figures and Tables

**Table 1:** Baseline characteristics of the study population.

	Intestinal Resection < 3 Years		*p*-Value
	Yes (*n* = 46)	No (*n* = 291)	
Age, years *	42.65 ± 12.96	36.40 ± 11.77	0.001
Male (%)	37 (80.4)	206 (70.8)	0.142
Smoking (%)			0.216
Nonsmoker	32 (69.6)	227 (78.0)	
Former smoker	12 (26.1)	54 (18.6)	
Current smoker	2 (4.3)	10 (3.4)	
Family history of IBD; yes (%)	2 (4.3)	8 (2.7)	0.902
Follow up duration, years *	6.43 ± 4.63	9.09 ± 7.60	0.022
CDAI at diagnosis *	62.87 ± 57.59	57.46 ± 81.35	0.700
Location (%)			0.070
Ileum	15 (32.6)	100 (34.4)	
Colon	5 (10.9)	39 (13.4)	
Ileocolon	26 (56.5)	152 (52.2)	
+ Upper GI ^†^	3 (6.5)	43 (14.8)	
Behavior (%)			0.013
Non-stricturing, non-penetrating	13 (28.2)	236 (81.1)	
Stricturing	8 (17.4)	29 (10.0)	
Penetrating	25 (54.4)	26 (8.9)	
+ Perianal disease ^†^	8 (17.4)	96 (33.0)	
Duration between diagnosis and surgery, years *	0.38 ± 0.60	7.78 ± 3.23	<0.0001
Medication (%)			
5-ASA	29 (63.0)	183 (62.9)	0.993
Steroid	23 (50.0)	161 (55.3)	0.279
Immunomodulator	28 (60.9)	190 (65.3)	0.746
Anti-Tumor necrosis factor	23 (50.0)	151 (51.9)	0.944
Other biologics	2 (4.3)	15 (5.2)	0.757

5-ASA, 5-aminosalicylate; CDAI, Crohn’s disease activity index; GI, gastrointestinal; IBD, inflammatory bowel disease; *n*, number. * Represented by the mean ± standard deviation. ^†^ Upper GI involvement and perianal disease were added as modifiers.

**Table 2:** Comparison of model performance with various feature combinations.

	Behavior Only *	Clinical Only ^†^	Clinical + Genotypes; Initial Model ^‡^	Clinical + Genotypes; Final Model (CatBoost and SHAP) ^§^
Clinical features (*n*)	1	7	7	2
Genotypes (*n*)	0	0	102	4
Internal validation AUROC	0.868	0.782	0.552	0.878
External validation AUROC	0.457	0.805	0.737	0.835

All models were tuned for hyperparameters through Bayesian optimization. *n*, number; AUROC, area under the receiver operating characteristic. * Behavior is a feature classified according to the Montreal classification that directly affects the prognosis of the patient. ^†^ Performance of a prediction model using only clinical information. ^‡^ The result of using seven clinical features and 102 single-nucleotide polymorphisms (SNPs) obtained as a result of genome-wide association study (GWAS) preselection. ^§^ Our model obtained as a result of feature selection.

**Table 3:** Comparison of model performance with various criteria of early intestinal resection.

Timing of Intestinal Resection		<1 Year	<3 Years	<5 Years	Any Period
Internal test set (case:control, *n*)		7:44	8:43	11:40	14:37
External test set (case:control, *n*)		15:111	19:107	21:105	32:94
AUROC		Under 1 year	Under 3 years	Under 5 years	Above 5 years
Internal test	Clinical-only	0.860	0.782	0.696	0.707
	Clinical + genotype; Final model	0.953	0.878	0.739	0.760
External test	Clinical-only	0.850	0.805	0.780	0.771
	Clinical + genotype; Final model	0.884	0.835	0.802	0.792

*n*, number.

## Data Availability

The corresponding author had full access to all data and takes full responsibility for the veracity of the data and analysis.

## Supplementary Materials

The following are available online at https://www.mdpi.com/2077-0383/10/4/633/s1. Table S1, Dataset including discovery set and external validation for machine learning, Table S2, Baseline characteristics of the training set and internal validation set, Table S3, Selected clinical and genetic features for a prediction model.

## Abbreviations

AUROC, area under the receiver operating characteristic; CD, Crohn’s disease; CRP, C-reactive protein; CV, cross-validation; FSTL5, follistatin-like 5; GIGYF2, Growth Factor Receptor Bound Protein 10-interacting GYF protein 2; GWAS, genome-wide association study; IBD, inflammatory bowel disease; LD, linkage disequilibrium; MMP, matrix metalloproteinase; OR, odds ratio; PD, Parkinson’s disease; SNPs, single-nucleotide polymorphisms; TNF, tumor necrosis factor; QC, quality control; SHAP, Shapley Additive Explanations.
