# Supplementary material for: Development of a Clinical and Genetic Prediction Model for Early Intestinal Resection in Patients with Crohn’s Disease: Results from the IMPACT Study
Source: J Clin Med. 2021 Feb 7;10(4):633. doi: 10.3390/jcm10040633 (PMC7915022; doi:10.3390/jcm10040633)
Supplement: Supplementary file 1 [file jcm-10-00633-s001.pdf]

Supplementary Table S1. Dataset including discovery set and external validation for machine learning

Dataset	Case, n (%)	Control, n (%)	Total, n	Usage
Discovery set	46 (14%)	291 (86%)	337	
Training set	38 (13%)	248 (87%)	286	GWAS, CatBoost, SHAP
Internal validation set	8 (16%)	43 (84%)	51	model performance
External validation set	19 (15%)	107 (85%)	126	model performance

n, number

Supplementary Table S2. Baseline characteristics of the training set and internal validation set

Training set (n = 286)	Intestinal resection < 3 years		
	Yes (n = 38)	No (n = 248)	*P*-value
Age, years *	43.63 ± 13.48	36.49 ± 11.90	0.001
Male (%)	30 (78.9)	174 (70.2)	0.533
Smoking (%)			0.165
Nonsmoker	25 (65.8)	194 (78.2)	
Former smoker	12 (31.6)	53 (21.4)	
Current smoker	1 (2.6)	1 (0.4)	
Family history of IBD; yes (%)	2 (5.3)	8 (3.2)	0.954
Follow up duration, years *	5.45 ± 3.61	8.93 ± 5.02	< .0001
CDAI at diagnosis *	66.36 ± 56.59	53.07 ± 75.61	0.353
Location (%)			0.010
Ileum	13 (34.2)	63 (25.4)	
Colon	3 (7.9)	37 (14.9)	
Ileocolon	22 (57.9)	148 (59.7)	
+ Upper GI †	0 (0.0)	25 (10.1)	
Behavior (%)			0.252
Non-stricturing, non-penetrating	11 (28.9)	199 (80.2)	
Stricturing	6 (15.8)	27 (10.9)	
Penetrating	21 (55.3)	22 (8.9)	
+ Perianal disease †	5 (13.2)	80 (32.3)	
Duration between diagnosis and surgery, years *	0.48 ± 0.71	8.13 ± 3.24	< .0001
Medication (%)			
5-ASA	24 (63.2)	150 (60.5)	0.856
Steroid	20 (52.6)	141 (56.9)	0.419
Immunomodulator	25 (65.8)	162 (65.3)	0.832
Anti-TNF	17 (44.7)	127 (51.2)	0.161
Other biologics	2 (5.3)	13 (5.2)	0.103
Internal validation set (n = 51)	Intestinal resection < 3 years		
	Yes (n = 8)	No (n = 43)	*P*-value

Age, years *	38.2 ± 10.50	35.93 ± 11.06	0.557
Male (%)	7 (87.5)	32 (74.4)	0.343
Smoking (%)			0.959
Nonsmoker	7 (87.5)	33 (72.1)	
Former smoker	0 (0.0)	1 (7.0)	
Current smoker	1 (12.5)	9 (20.9)	
Family history of IBD; yes (%)	0 (0.0)	0 (2.7)	
Follow up duration, years *	9.19 ± 7.48	10.35 ± 15.88	0.743
CDAI at diagnosis *	36.77 ± 69.01	83.10 ± 106.75	0.359
Location (%)			0.896
Ileum	2 (25.0)	37 (86.0)	
Colon	2 (25.0)	2 (4.7)	
Ileocolon	4 (50.0)	4 (9.3)	
+ Upper GI †	3 (37.5)	18 (37.3)	
Behavior (%)			0.034
Non-stricturing, non-penetrating	2 (25.0)	37 (86.0)	
Stricturing	2 (25.0)	2 (4.7)	
Penetrating	4 (50.0)	4 (9.3)	
+ Perianal disease †	3 (37.5)	16 (37.2)	
Duration between diagnosis and surgery, years *	0.52 ± 0.92	5.16 ± 0.69	< .0001
Medication (%)			
5-ASA	5 (62.5)	33 (76.7)	0.397
Steroid	3 (37.5)	20 (46.5)	
Immunomodulator	3 (37.5)	28 (65.1)	0.084
Anti-TNF	6 (75.0)	24 (55.8)	0.456
Other biologics	0 (0.0)	2 (4.7)	

5-ASA, 5-aminosalicylate; CDAI, Crohn's disease activity index; GI, gastrointestinal; IBD, inflammatory bowel disease; n, number

* Represented by mean ± standard deviation

† Upper GI involvement and perianal disease are added as modifiers

Supplementary Table S3. Selected clinical and genetic features for a prediction model

Features	Training set			Internal Validation Set			External Validation Set		
Age (mean) *		26.97			26.02			24.61	
Behavior (n)									
NS+NP		210			39			75	
Stricturing		33			4			31	
Penetrating		43			8			20	
SNPs (n) †	Major homo	Hetero zygote	Minor homo	Major homo	Hetero zygote	Minor homo	Major homo	Hetero zygote	Minor homo
rs28785174 (FSTL5)	151	113	22	30	19	2	67	53	6
rs7660164 (FSTL5)	167	102	17	33	17	1	79	41	6
rs60532570 (GIGYF2)	205	76	5	40	9	2	88	31	7
rs13056955 (None)	133	115	38	26	18	7	61	45	20

n, number; NS, non-stricturing; NP, non-penetrating; homo, homozygote

* Mean ± standard deviation

† Selective SNPs and comparable genes are shown. The proportions of major homozygotes, heterozygotes, and minor homozygotes are presented.
